# Whole-Genome Transformation of Yeast Promotes Rare Host Mutations with a Single Causative SNP Enhancing Acetic Acid Tolerance

**DOI:** 10.1128/mcb.00560-21

**Published:** 2022-03-21

**Authors:** Marija Stojiljković, Arne Claes, Quinten Deparis, Mekonnen M. Demeke, Ana Subotić, María R. Foulquié-Moreno, Johan M. Thevelein

**Affiliations:** a Laboratory of Molecular Cell Biology, Institute of Botany and Microbiology, Department of Biology, KU Leuven, Leuven, Belgium; b Center for Microbiology, VIB, Leuven, Belgium; c NovelYeast bv, Open Bio-Incubator, Erasmus High School, Brussels, Belgium

**Keywords:** whole-genome transformation, whole-genome sequence analysis, allele exchange, industrial yeast, acetic acid tolerance, SNF4

## Abstract

Whole-genome (WG) transformation (WGT) with DNA from the same or another species has been used to obtain strains with superior traits. Very few examples have been reported in eukaryotes—most apparently involving integration of large fragments of foreign DNA into the host genome. We show that WGT of a haploid acetic acid-sensitive Saccharomyces cerevisiae strain with DNA from a tolerant strain, but not from nontolerant strains, generated many tolerant transformants, some of which were stable upon subculturing under nonselective conditions. The most tolerant stable transformant contained no foreign DNA but only seven nonsynonymous single nucleotide polymorphisms (SNPs), of which none was present in the donor genome. The *SNF4* mutation c.[805G→T], generating Snf4^E269^*, was the main causative SNP. Allele exchange of *SNF4*^E269^* or *snf4Δ* in industrial strains with unrelated genetic backgrounds enhanced acetic acid tolerance during fermentation under industrially relevant conditions. Our work reveals a surprisingly small number of mutations introduced by WGT, which do not bear any sequence relatedness to the genomic DNA (gDNA) of the donor organism, including the causative mutation. Spontaneous mutagenesis under protection of a transient donor gDNA fragment, maintained as extrachromosomal circular DNA (eccDNA), might provide an explanation. Support for this mechanism was obtained by transformation with genomic DNA of a yeast strain containing NatMX and selection on medium with nourseothricin. Seven transformants were obtained that gradually lost their nourseothricin resistance upon subculturing in nonselective medium. Our work shows that WGT is an efficient strategy for rapidly generating and identifying superior alleles capable of improving selectable traits of interest in industrial yeast strains.

## INTRODUCTION

Whole-genome (WG) transformation (WGT) with DNA from other strains or other species has been used occasionally to generate mutants with selectable phenotypes. A well-known application is the identification of penicillin resistance mutations in clinical isolates of Streptococcus pneumoniae by WGT of sensitive S. pneumoniae strains with a known genetic background, using genomic DNA (gDNA) derived from the clinical isolates coupled with next-generation sequencing (NGS) of the resulting antibiotic-resistant transformants (1). Similar experiments have been performed on linezolid resistance mutations in S. pneumoniae (2) and paromomycin resistance in *Leishmania* parasites (3). Detailed analysis of transferred polymorphisms in Haemophilus influenzae whole-genome transformants revealed incorporation of multiple heterologous fragments 5 to 10 kb in length (4). Sequence similarity is a major prerequisite for successful integration of heterologous genomic DNA fragments (5).

In eukaryotes, on the other hand, very few publications have dealt with WGT, and virtually nothing is known about the genetic basis of superior traits established in such whole-genome transformants. Recursive WGT with gDNA of Saccharomyces cerevisiae and Pichia stipitis was used to obtain a hybrid strain displaying efficient xylose fermentation as well as high ethanol tolerance (6). In this case, integration of complete genes encoding functional xylose metabolism enzymes must have happened to allow for selection of transformants growing on xylose. The resulting hybrid strain displayed a mixture of traits from the two parent strains, apparently due to a mixed genomic constitution. The procedure was considered a form of accelerated genome shuffling by the authors (6). WGT of rice callus with gDNA from wild rice (Zizania palustris) and a plasmid containing a gene conferring hygromycin resistance allowed recovery under selection of transgenic plants with grain characteristics from wild rice. Amplified fragment length polymorphism (AFLP) analysis suggested that a significant amount of DNA from *Zizania* had been introduced (7). From these examples, it seems to have been concluded generally that WGT causes integration of large fragments of foreign DNA in a random, uncontrolled manner. As a result, there is a complete lack of information on the causative genetic changes responsible for altered phenotypes after WGT, irrespective of the changes introduced in the genome. Although useful genes can be introduced in this way, the excess of foreign DNA thought to be integrated would make genetic analysis cumbersome and could easily affect other properties in the organism, and in particular compromise other traits of commercial importance in, for instance, industrial microorganisms or crop plants. This has apparently made WGT a rather unattractive methodology for mutagenesis studies and for development of improved industrial organisms. Recent work from our group has applied WGT for improvement of thermotolerance and 5-hydroxymethylfurfural (HMF) tolerance in industrial yeast strains. In both cases, the WG transformants did not contain any foreign DNA, but just a very low number of single nucleotide polymorphisms (SNPs) compared to the parent strain, and the single causative SNP identified was also absent from the genome of the donor strain. In spite of this, WGT with genomic DNA from a strain with superior thermotolerance or HMF tolerance was essential to obtain stable WG transformants with improved tolerance (8, 9).

Acetic acid tolerance is a trait of major importance in the food industry and in industrial yeast fermentations. Acetic acid has high toxicity and is commonly used in the food industry as an antimicrobial preservative (10). At low pH, the protonated form can easily diffuse through membranes and drastically lower the internal pH of cells and organelles, causing widespread inhibition of many cellular functions (11, 12). Acetic acid tolerance is particularly important in the production of bioethanol with genetically engineered yeast, using so-called second-generation substrates: i.e., hydrolysates of lignocellulosic biomass derived from waste streams or bioenergy crops (13, 14). Lignocellulose fibrils contain large numbers of acetyl groups, which are released during pretreatment and enzymatic hydrolysis and accumulate to high levels in the medium. Together with multiple other inhibitors being present as well as the ethanol produced, the acetic acid inhibits the fermentation process and especially the artificially engineered capacity of xylose fermentation (15, 16). Also, in bioethanol production with starch from food crops, sugar cane, or molasses (so-called first-generation substrates), acetic acid produced by contaminating acetic acid bacteria can accumulate to high levels, especially by water recycling practices, and cause significant inhibition of yeast fermentation (17). Sourdough preparation is another example in which yeast fermentation is likely compromised by the acetic acid produced in the preceding bacterial fermentation (18).

Many approaches have been used to improve the tolerance of yeast in ethanol production processes, including tolerance to acetic acid (19). A major component conferring acetic acid tolerance is the Haa1 transcription factor, and its overexpression or modification is well known to enhance acetic acid tolerance (20–24). Polygenic analysis of a yeast strain with high acetic acid tolerance confirmed the importance of *HAA1* and revealed *GLO1*, *DOT5*, *CUP2*, and *VMA7* as additional causative factors, but also suggested the existence of many other factors involved in high acetic acid tolerance (25). Overexpression of *WHI2* (26), *ACS2* (27), *RTC3* and *ANB1* (28), *ASC1*, and *GND1* and deletion of 50 different genes (29) were also shown to enhance acetic acid tolerance. Evolutionary adaptation in media with increasing acetic acid levels is well known to generate strains with higher acetic acid tolerance, although poor stability and the presence of compromising background mutations are possible drawbacks (30–32). Evolutionary adaptation with conditions alternating between the presence and absence of acetic acid was successful in generating stable strains and led to identification of *ASG1*, *ADH3*, *SKS1*, and *GIS4* as novel causative genes (33). Multiple genomic approaches have been applied to analyze the genetic basis of acetic acid tolerance as well as the adaptive stress response to acetic acid in yeast, but identification of efficient gene tools to enhance acetic acid tolerance in a predictable manner in industrial yeast strains from such studies has been very limited (34, 35).

Most research on acetic acid tolerance in S. cerevisiae has used different laboratory strain backgrounds with various levels of inherent acetic acid tolerance, as well as single genetic modifications. It is likely that combination of genetic modifications will lead to higher acetic acid tolerance, but no systematic studies in this respect have been performed, and it is thus unclear what the maximal acetic acid tolerance is that could be reached in S. cerevisiae by combining multiple genetic modifications present in different natural strains. It is interesting to note that the yeast Zygosaccharomyces baillii displays much higher inherent acetic acid tolerance than even the best S. cerevisiae strains. It is unclear what genetic factors are responsible for this very high tolerance, although recent results highlight the possible importance of sphingolipid content (36, 37).

The aim of this work was to use intraspecies WGT to transfer novel acetic acid tolerance mutations present in a strain with superior acetic acid tolerance into a sensitive host strain, similar to the intraspecies transfer of antibiotic tolerance from tolerant to sensitive bacterial strains (1, 2). Among the many transformants with higher acetic acid tolerance obtained, the most tolerant strain was analyzed in detail. It contained a surprisingly low number of SNPs, of which only one, *SNF4*^E269^*, was clearly causative. It also improved acetic acid tolerance strongly in unrelated industrial strain backgrounds. Since this mutation was absent in the genome of the donor strain and since the genomic DNA of the host strain or other non-acetic acid-tolerant strains was unable to generate transformants with higher acetic acid tolerance, the genomic DNA of the donor strain in some way appeared to support the generation in the host strain of a spontaneous mutation conferring higher acetic acid tolerance in the presence of very few additional background mutations. Our results reveal that WGT can be used to improve selectable traits in yeast cells, with a low risk of causing side effects, and raise intriguing questions as to the molecular mechanism involved in generating the novel, selectable mutations conferring higher fitness.

## RESULTS

### Isolation of whole-genome transformants.

We have identified in our S. cerevisiae strain collection a sake strain, K11, with high acetic acid tolerance during fermentation, in spite of the fact that it contained all inferior alleles of the five genes previously identified by polygenic analysis as causative elements for high acetic acid tolerance in strain JT22689 (PYCC 4542) (25). Hence, K11 should contain novel genetic elements determining high acetic acid tolerance. We decided to use whole-genome transformation to identify novel causative alleles in strain K11. As the host strain, we used strain ER18A, a haploid derivative of the industrial strain Ethanol Red, widely used for first-generation bioethanol production and displaying relatively low acetic acid tolerance (25).

**FIG 1:**
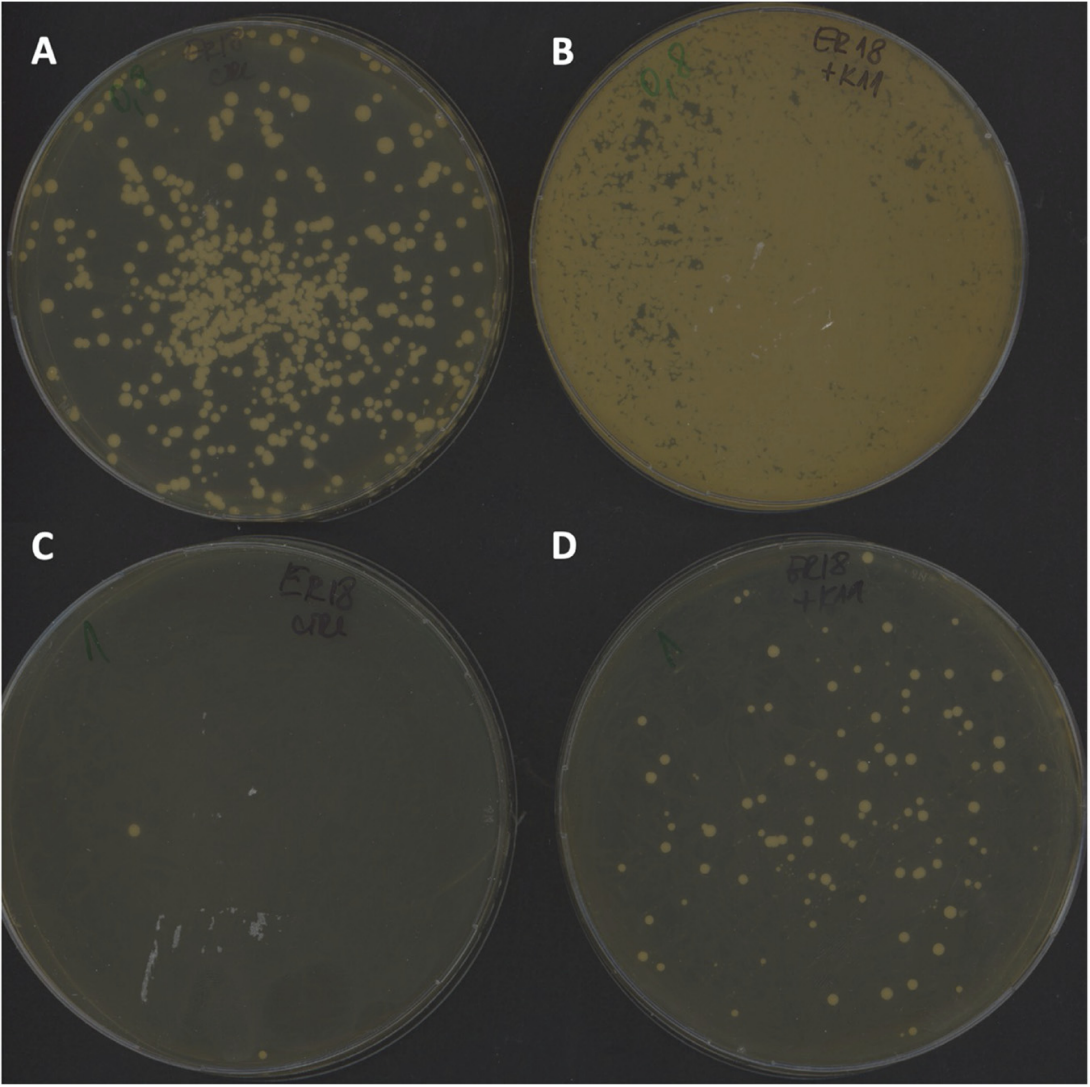
Isolation of WG transformants on solid nutrient medium. The two upper plates show selection in the presence of 8 g/L acetic acid. (A) Control plate: ER18A transformed with water. (B) ER18A transformed with gDNA of K11. The two lower plates show selection in the presence of 10 g/L acetic acid. (C) Control plate: ER18A transformed with water. (D) ER18A transformed with gDNA of K11.

After standard electroporation transformation of the recipient acetic acid-sensitive strain ER18A with gDNA of the acetic acid-tolerant strain K11 and selection on yeast extract-peptone-dextrose (YPD) plates with different levels of acetic acid from 5 g/L to 12 g/L at pH 4.7 (which is just below the pK_a_ of 4.76 for acetic acid to ensure stringent conditions), 56 independent transformants were obtained in the presence of 10 g/L acetic acid, of which 53 showed stable acetic acid tolerance after subculturing on YPD plates (Table 1 and Fig. 1). In the presence of lower acetic acid concentrations, the plates were overgrown with colonies, and in the presence of 12 g/L acetic acid, only one unstable transformant was obtained. When strain ER18A was transformed with only water or with gDNA from ER18A itself, the number of transformant colonies was much lower, and all those tested were unstable (Table 1). The WG transformation and selection procedure was repeated, and each time, stable acetic acid-tolerant transformants were obtained only with gDNA from an acetic acid-tolerant strain and not with gDNA from a nontolerant strain.

**TABLE 1:** Number of transformants obtained after WGT of ER18A with either water or gDNA from ER18A itself or from acetic acid tolerant strain K11

Acetic acid concn (g/L)	Result for host strain + gDNA source shown^*a*^					
	ER18A + H_2_O		ER18A + ER18A		ER18A + K11	
	Total colonies	Stable colonies	Total colonies	Stable colonies	Total colonies	Stable colonies
5	Full plate	NA	Full plate	NA	Full plate	NA
6	Full plate	NA	Full plate	NA	Full plate	NA
8	±100 colonies	NT	±100 colonies	NT	Full plate	NA
10	2	0	3	0	56	53
12	0	NA	0	NA	1	0

a NA, not applicable; NT, not tested.

**FIG 2:**
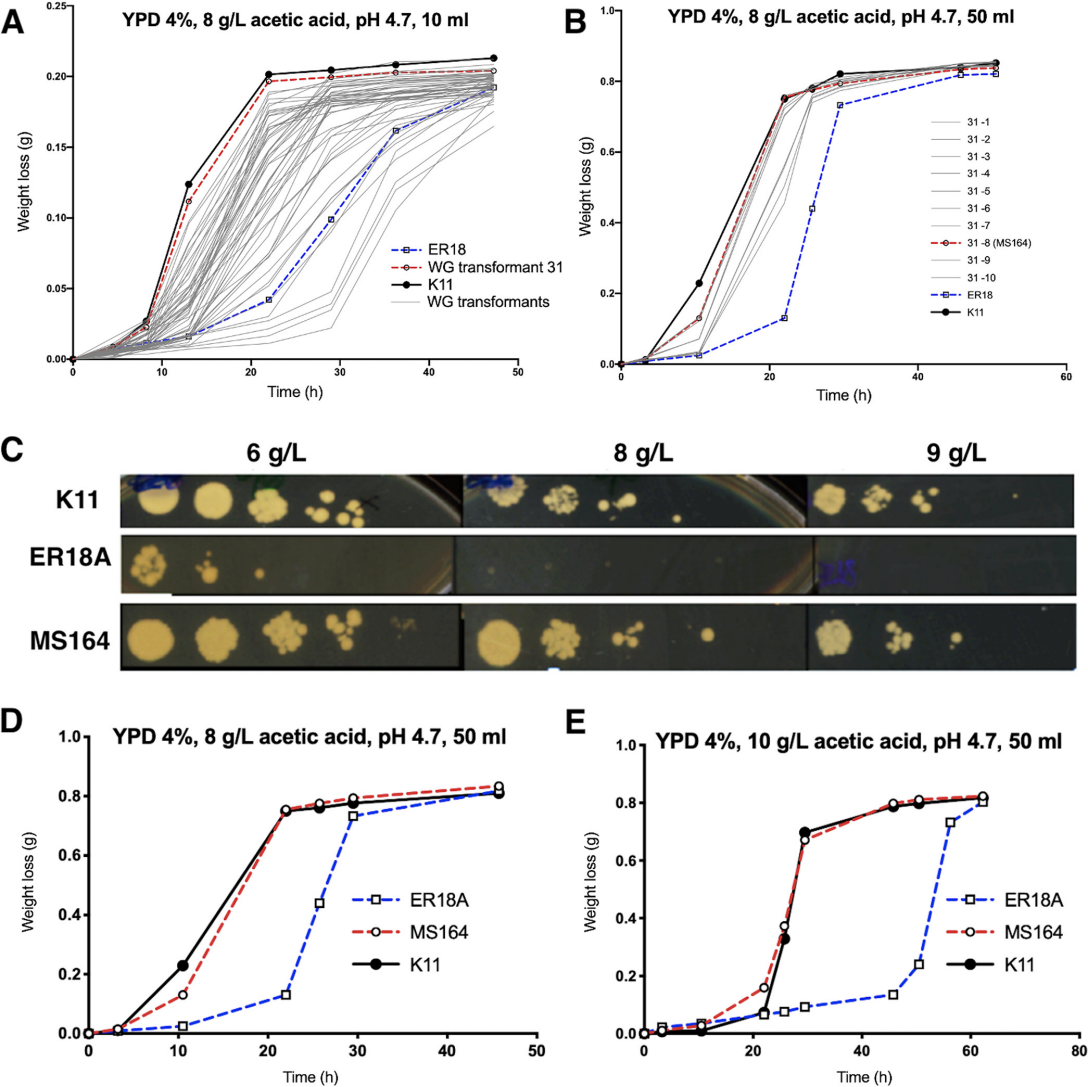
Selection of the WG transformant 31-8 (MS164) as most tolerant to acetic acid. (A) Fermentation performance of the 53 stable WG transformants in 10-mL fermentations with YPD containing 4% glucose plus 8 g/L acetic acid at pH 4.7. The starting OD_600_ was 2.0. (B) Fermentation performance of 10 single colonies of WG transformant 31 in 50-mL fermentations with YPD containing 4% glucose plus 8 g/L acetic acid at pH 4.7. The starting OD_600_ was 2.0. (C) Spot assay for acetic acid tolerance. The strains K11 (gDNA donor strain), ER18A (parent strain), and MS164 were grown in YPD until they reached an OD_600_ of 1.0, and 1:10 serial dilutions were spotted onto YPD nutrient plates in the presence of 6, 8, or 9 g/L acetic acid. (D and E) Fermentation performance of ER18A, MS164, and K11 in the presence of 8 g/L or 10 g/L acetic acid, as indicated.

The 53 stable transformants were tested in 10-mL small-scale fermentations at a standard temperature of 35°C and showed a wide range of acetic acid tolerances, clearly different from that of the parent strain ER18A (Fig. 2A). This is consistent with the notion that the WG transformation procedure led to the generation and selection of a range of different mutations. Single isolates of colony 31, which showed the best fermentation performance in liquid cultures and also the best growth performance on solid nutrient plates in the presence of acetic acid, were evaluated in 50-mL small-scale fermentations. Isolate 31-8 showed the highest and most reproducible acetic acid tolerance, also after storage of the strain at −80°C (Fig. 2B). This isolate was called MS164 and was selected for further analysis. Strain MS164 showed clear improvement in growth also on solid YPD medium supplemented with 9g/L acetic acid at pH 4.7, whereas its parent strain, ER18A, only grew up to 6g/L acetic acid (Fig. 2C). Furthermore, MS164 also displayed better performance than ER18A in 50-mL small-scale fermentations in the presence of different concentrations of acetic acid. Its performance was comparable to that of strain K11, from which the gDNA was derived (Fig. 2D).

### Whole-genome sequence analysis of SNPs in transformant MS164.

Transformant MS164 was submitted to whole-genome sequence analysis to identify the genetic changes introduced by transformation of the host strain, ER18A. The complete genome sequences, including intergenic regions, of both strains were aligned and compared. Unexpectedly, MS164 and ER18A only differed in 12 single nucleotide polymorphisms (SNPs), of which 5 were synonymous mutations, while the other 7 were nonsynonymous mutations (Table 2). Surprisingly, none of the seven nonsynonymous SNPs was present in the genome of donor strain K11. Also, none of these SNPs was present in 1,011 whole-genome-sequenced S. cerevisiae strains (38), except for SNP3, which was present in 7 strains (Table 2).

**TABLE 2:** The seven nonsynonymous and intergenic SNPs introduced during whole-genome transformation in the ER18A host strain

SNP no.	Chromosome	Nucleotide in strain			Gene	Nucleotide change in ORF	Amino acid change in protein	No. present in other S. cerevisiae strains
		ER18A parent	MS164 transformant	K11 gDNA donor				
1	VII	G	T	G	*SNF4*	c.[805G→T]	Snf4^E269^*	0/1,011
2	XIV	C	A	G	Intergenic region	[579C→A] downstream from start codon of *FKH2* on Watson strand		0/1,011
3	IV	C	T	C	*SEC31*	c.[2267C→T]	Sec31^A756V^	7/1,011
4	IV	C	A	C	*PPN1*	c.[227C→A]	Ppn1^P76H^	0/1,011
5	X	G	A	G	*YJR120W*	c.[46G→A]	Yjr120w^A16T^	0/1,011
6	XI	C	T	C	*TCD2*	c.[1051C→T]	Tcd2^P351S^	0/1,011
7	XIII	G	A	G	*STB4*	c.[2285G→A]	Stb4^C762Y^	0/1,011

### Identification of the causative SNP by allele exchange.

**FIG 3:**
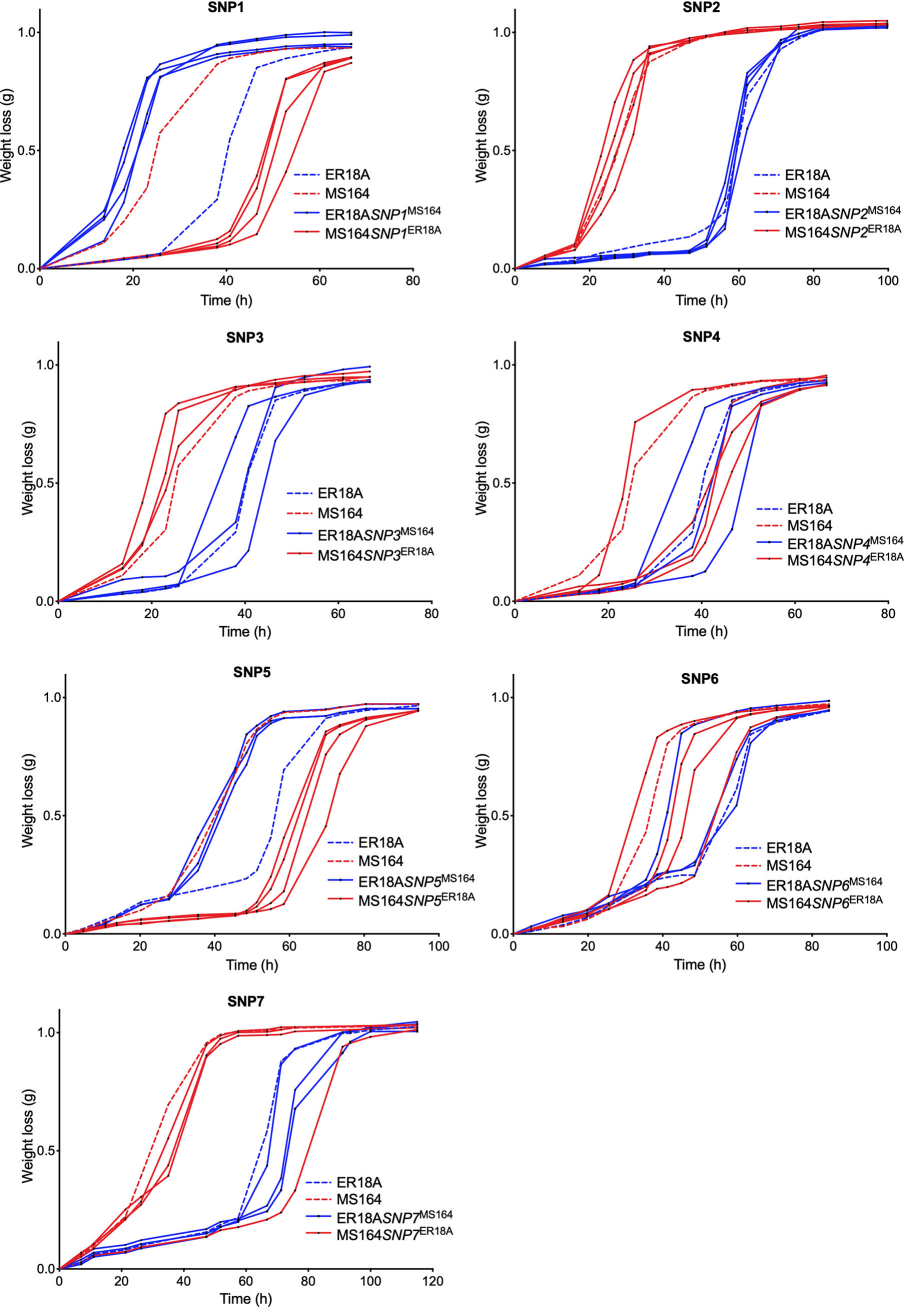
Fermentation performance of the original ER18A parent strain, ER18A derivatives engineered for each SNP, the MS164 transformant, and MS164 derivatives reverse engineered for each SNP in the presence of acetic acid. The control strains are indicated with dashed lines: ER18A in blue and MS164 in red. All SNP-engineered strains are indicated with full lines: the downgraded derivatives of MS164 in red (3 or 4 replicates) and the upgraded derivatives of ER18A in blue (3 or 4 replicates). Fermentations were performed at 35°C, with constant stirring at 120 rpm, at pH 4.7 in 50 mL YPD medium with 40 g/L glucose and supplemented with 10 g/L acetic acid.

We assessed the relevance of all seven nonsynonymous SNPs individually for high acetic acid tolerance by allele exchange between the ER18A and MS164 strains by using the CRISPR/Cas9 methodology. The nucleotide from the inferior ER18A host strain was exchanged into the corresponding position in the MS164 strain to assess whether it downgraded acetic acid tolerance, and the nucleotide from the superior transformant MS164 was exchanged into the inferior ER18A strain to assess whether it upgraded acetic acid tolerance. The resulting strains were evaluated for fermentation performance in YPD medium containing 10 g/L acetic acid with at least three biological replicates. The results are shown in Fig. 3.

We observed large differences in the length of the lag phase between technical and biological replicates. In our experience, there are two causes for this: (i) the harsh conditions at the edge of viability are influenced by the high acetic acid levels at a pH below acetic acid’s pK_a_, and (ii) our working pH is very close to the pK_a_ of acetic acid, and therefore even slight variations in medium pH can greatly contribute to the variations in the ratio of the dissociated versus undissociated form and thus to the degree of toxicity. Another reason might be differences between the replicates in the level of adaptation to the high acetic acid level during the lag phase (24). Hence, all replicates are shown as individual fermentations rather than an average from different replicates with standard deviation. For an SNP to be considered causative for high acetic acid tolerance, all replicates of the upgraded ER18A strain should ferment better than the inferior parent, ER18A, while all the replicates of the downgraded MS164 strain should ferment worse than the superior parent, MS164.

**FIG 4:**
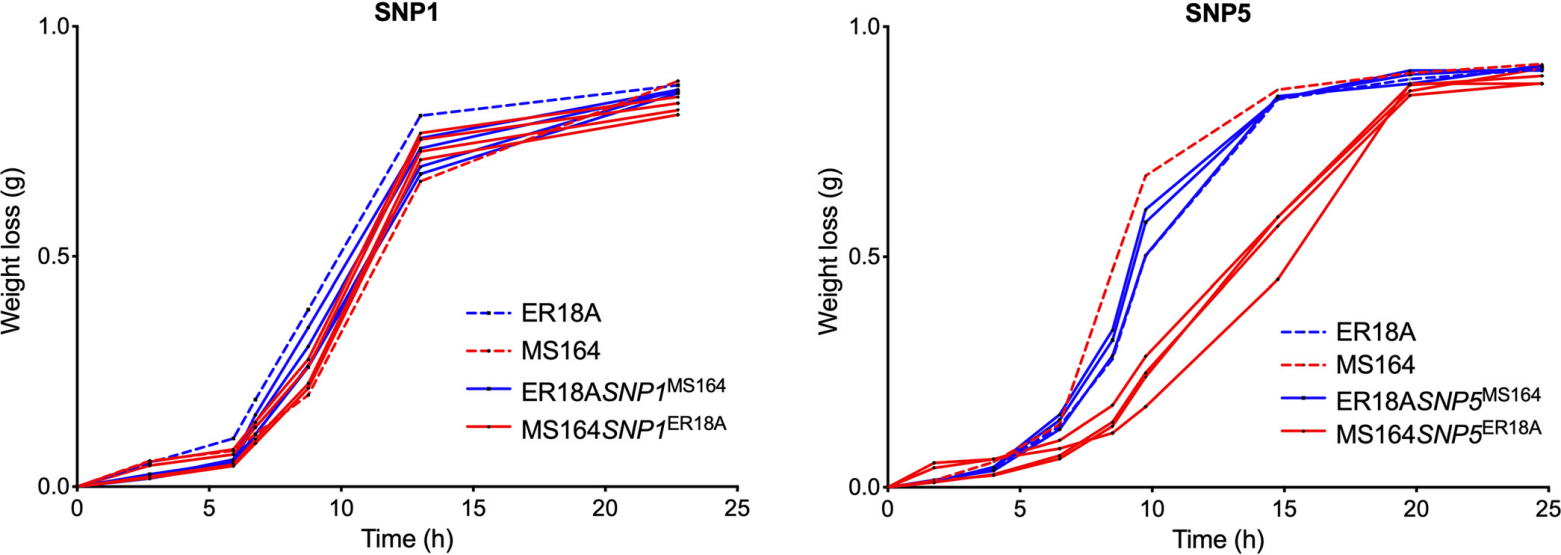
Fermentation performance of the original ER18A parent strain, ER18A derivatives engineered for individual SNP1 or SNP5, the MS164 transformant, and MS164 derivatives reverse engineered for individual SNP1 or SNP5, in the absence of acetic acid. The control strains are indicated with dashed lines: ER18A in blue and MS164 in red. All SNP-engineered strains are indicated with full lines: the downgraded derivatives of MS164 in red (3 or 4 replicates) and the upgraded derivatives of ER18A in blue (3 or 4 replicates). Fermentations were performed at 35°C, with constant stirring at 120 rpm, in 50 mL YPD medium with 40 g/L glucose (and no acetic acid addition).

After assessing all 7 SNPs individually, we can conclude that only SNP1 and SNP5 had a major effect on fermentation performance in the presence of acetic acid. To distinguish whether the effect of the SNP was truly due to improvement of acetic acid tolerance or whether it affected fermentation capacity itself, we tested the strains also under the same conditions in YPD medium in the absence of acetic acid (Fig. 4).

The results showed that SNP1 exchange had no effect in the absence of acetic acid. On the other hand, the downgrading of MS164 for SNP5 caused a reduction in the fermentation rate in the absence of acetic acid. The upgrading of ER18A for SNP5 did not have a significant effect on the fermentation rate. Upgrading SNP5 in other genetic backgrounds gave variable effects on fermentation performance in the presence of acetic acid, ranging from worsening, to little or no significant effect, to improvement. For instance, upgrading the two copies of *YJR120W* in the diploidized ER18A strain did not improve performance. Because of the apparent side effect on the growth rate of MS164 with the engineered SNP5 and the variable effects of SNP5 upgrading in other genetic backgrounds, we did not explore SNP5 further for improvement of acetic acid tolerance.

SNP1 is located in the *SNF4* open reading frame (ORF) at position 805, for which the host strain, ER18A, as well as the gDNA donor strain, K11, contain guanine (G), while the transformant MS164 has thymine (T). The modification from G to T leads to a change from glutamic acid^269^ into a stop codon, resulting in a truncated *SNF4* gene product. The wild-type Snf4 protein contains 323 amino acids, and the early stop codon results in a protein 54 amino acids shorter. We have resequenced the *SNF4* gene in the K11 isolate of our lab that we used for extraction of the genomic DNA, and it also did not contain the c.[805G→T] mutation resulting in Snf4^E269^*.

### Comparison of *snf4*^E269^* and *snf4*Δ strains for acetic acid tolerance.

**FIG 5:**
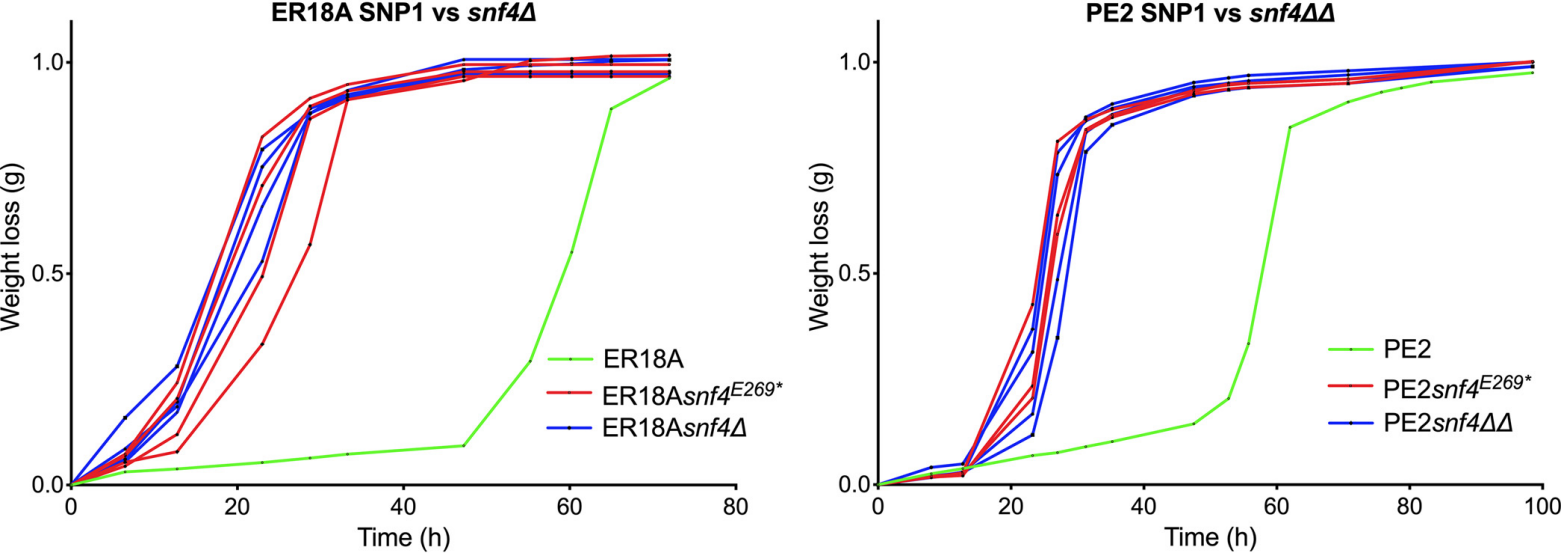
Fermentation performance of the strains ER18A, PE2, and ER18A or PE2 derivatives with engineered SNP1 (*snf4*^E269^*) or *snf4Δ* in all *SNF4* alleles present, in the presence of acetic acid. The strains are indicated by different colors: ER18A and PE2 in green, ER18A and PE2 derivatives with engineered SNP1 (*snf4*^E269^*) in all *SNF4* alleles present in red (3 or 4 replicates), and ER18A *snf4Δ* and PE2 *snf4ΔΔ* strains in blue (3 or 4 replicates). Fermentations were performed at 35°C, with constant stirring at 120 rpm, at pH 4.7 in 50 mL YPD medium with 40 g/L glucose and supplemented with 10 g/L (ER18A) or 11 g/L (PE2) acetic acid.

We also compared the effect of the *snf4*^E269^* nonsense mutation with that of *snf4Δ* on fermentation performance of ER18A in the presence of acetic acid (Fig. 5). The ER18A strain with the *SNF4*^E269^* mutation showed the same improvement of fermentation performance in the presence of acetic acid as the *snf4Δ* strain, suggesting that the truncated Snf4* protein is inactive. To evaluate the effect of *SNF4* modification in another industrial strain genetic background, we introduced two copies each of *SNF4*^E269^* or *snf4Δ* into the diploid Brazilian bioethanol strain PE2. In both cases, we observed a strong improvement of the fermentation rate in the presence of acetic acid, indicating that inactivation of Snf4 also improves acetic acid tolerance in other genetic backgrounds (Fig. 5). Single deletion of *SNF4* had no effect, indicating that the mutation is recessive.

**FIG 6:**
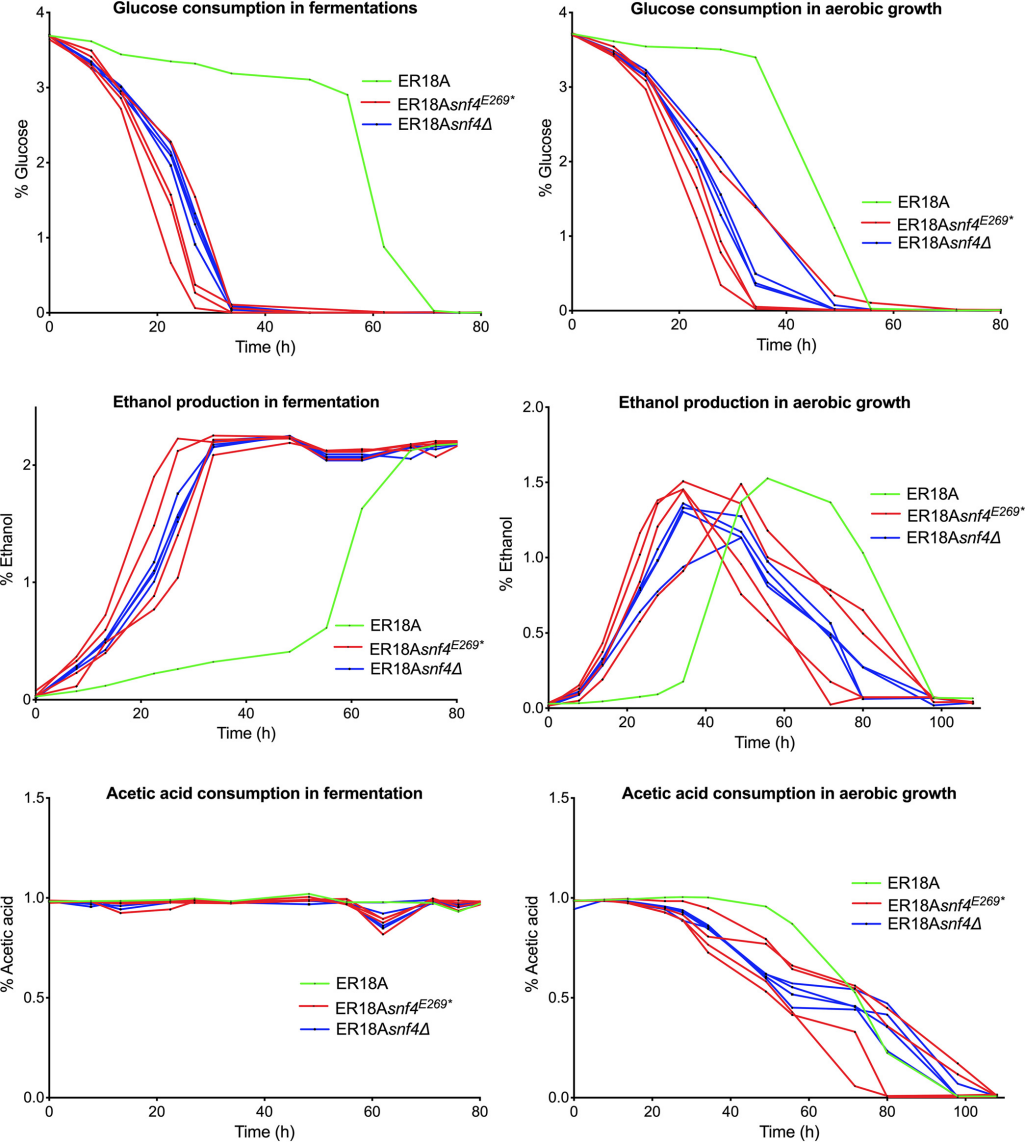
Glucose consumption, ethanol production, and acetic acid consumption during semianaerobic fermentation or aerobic growth of the strain ER18A and ER18A derivatives with engineered SNP1 (*snf4*^E269^*) in *SNF4* or *snf4Δ* in the presence of acetic acid. The strains are indicated by different colors: ER18A in green, ER18A derivatives with engineered SNP1 (*snf4*^E269^*) in *SNF4* in red (4 replicates), and ER18A *snf4Δ* in blue (4 replicates). Fermentations were performed at 35°C, with constant stirring at 120 rpm, at pH 4.7 in 50 mL YPD medium with 40 g/L glucose and supplemented with 10 g/L acetic acid. Growth assays under aerobic conditions were performed at 30°C, with constant shaking at 200 rpm, at pH 4.7 in 50 mL YPD medium with 40 g/L glucose and supplemented with 10 g/L acetic acid. Controls in the absence of acetic acid showed no effect of *snf4*^E269^* under this condition compared to the parent strain (Fig. 4).

Next, we evaluated whether the *snf4*^E269^* and *snf4Δ* genetic modifications affected other properties of the ER18A strain in the presence of acetic acid: glucose consumption and ethanol production during semianaerobic fermentations and during aerobic growth in shake flasks. YP medium with 40 g/L glucose and 10 g/L acetic acid at pH 4.7 and 30°C was used. Samples were taken every few hours, from both fermentation tubes and growth flasks, and analyzed by high-performance liquid chromatography (HPLC). The *snf4*^E269^* and *snf4Δ* engineered ER18A strains showed much faster glucose consumption and ethanol production both during semianaerobic fermentation and during aerobic growth (Fig. 6). The added acetic acid was only consumed during aerobic growth and not during semianaerobic fermentation. The *snf4*^E269^* and *snf4Δ* engineered ER18A strains initially showed faster acetic acid consumption than the ER18A strain, possibly due to their higher acetic acid tolerance, whereas later the difference from the control strain disappeared when the acetic acid had dropped to lower levels (Fig. 6). The results confirmed superior and similar performance of the *snf4*^E269^* and *snf4Δ* engineered ER18A strains compared to the control ER18A strain.

### Effect of *snf4*^E269^* and *snf4*Δ on growth under different conditions.

**FIG 7:**
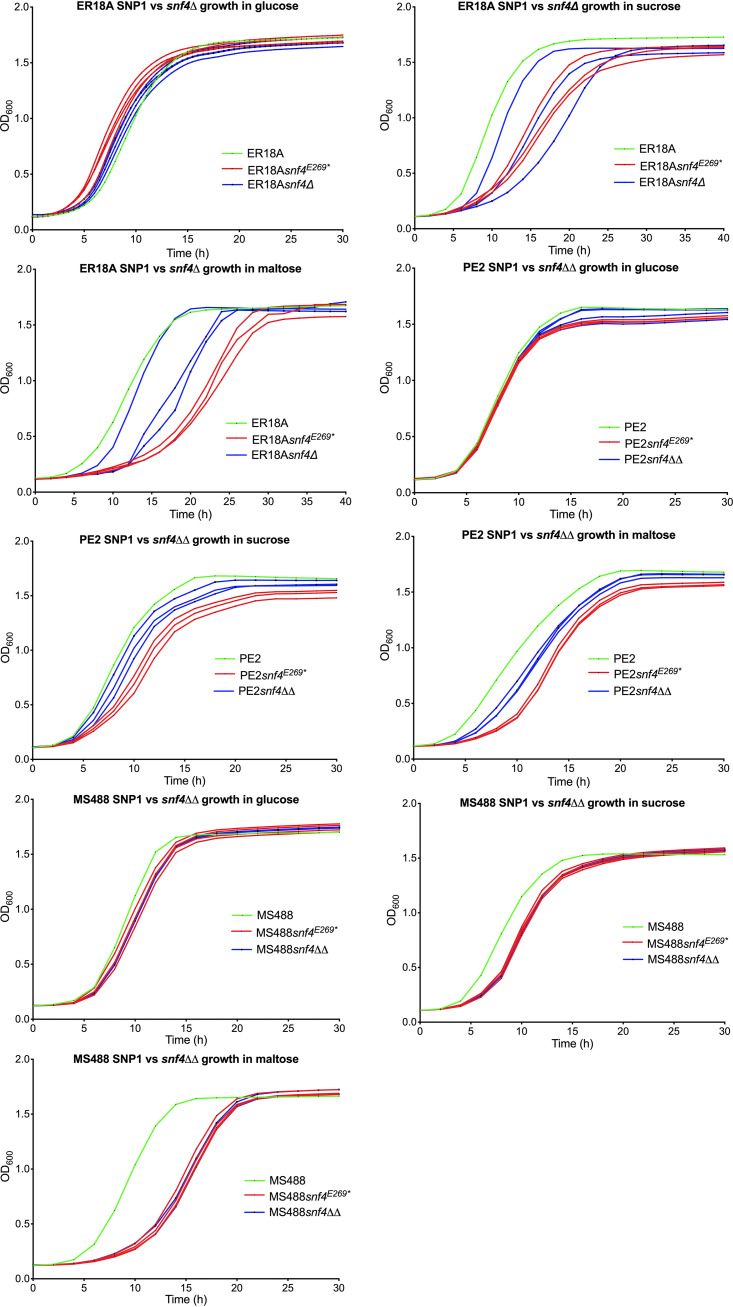
Growth assays in microtiter plate format using different carbon sources with the strains ER18A, PE2, MS488, and ER18A or PE2 or MS488 derivatives with engineered SNP1 (*snf4*^E269^*) or *snf4Δ* in all *SNF4* alleles present in the absence of acetic acid. The strains are indicated by different colors: ER18A, PE2, and MS488 in green, ER18A, PE2, and MS488 derivatives with engineered SNP1 (*snf4*^E269^*) in all *SNF4* alleles present in red (3 or 4 replicates), and ER18A *snf4Δ*, PE2 *snf4ΔΔ*, and MS488 *snf4ΔΔ* strains in blue (3 or 4 replicates). Growth assays were performed in microtiter plates in a Multiskan apparatus at 30°C, with intermittent shaking, in 200 μL YP medium with 40 g/L glucose, sucrose, or maltose (and absence of acetic acid). The maximum measurable OD_600_ in the Multiskan is ±2.0.

Snf4 is an activating subunit of the Snf1 protein kinase (“sucrose nonfermenting”) that is essential for sucrose utilization. However, mutations in *SNF1* were found to have pleiotropic effects also on utilization of other sugars, like galactose, raffinose, and maltose (39). *SNF4* mutants also showed pleiotropic defects in the utilization of carbon sources controlled by glucose repression and were compromised in producing secreted invertase (40). To test for additional effects of the *snf4*^E269^* and *snf4Δ* mutations, we measured growth on different carbon sources of the *snf4*^E269^* and *snf4Δ* (in all *SNF4* alleles present) strains in the ER18A, PE2, and MS488 (molasses strain JT28541 *HAA1*^S506N^) genetic backgrounds (Fig. 7). The utilization of glucose was not significantly affected by *snf4*^E269^* or *snf4Δ* in the three genetic backgrounds ER18A, PE2, and MS488. On the other hand, growth on sucrose and maltose was delayed to various extents in the three backgrounds (Fig. 7). This appears to be consistent with the role of Snf4 in glucose derepression. Strikingly, in most cases the *snf4*^E269^* point mutation, yielding truncated protein, had a more adverse effect than *snf4Δ* (Fig. 7).

**FIG 8:**
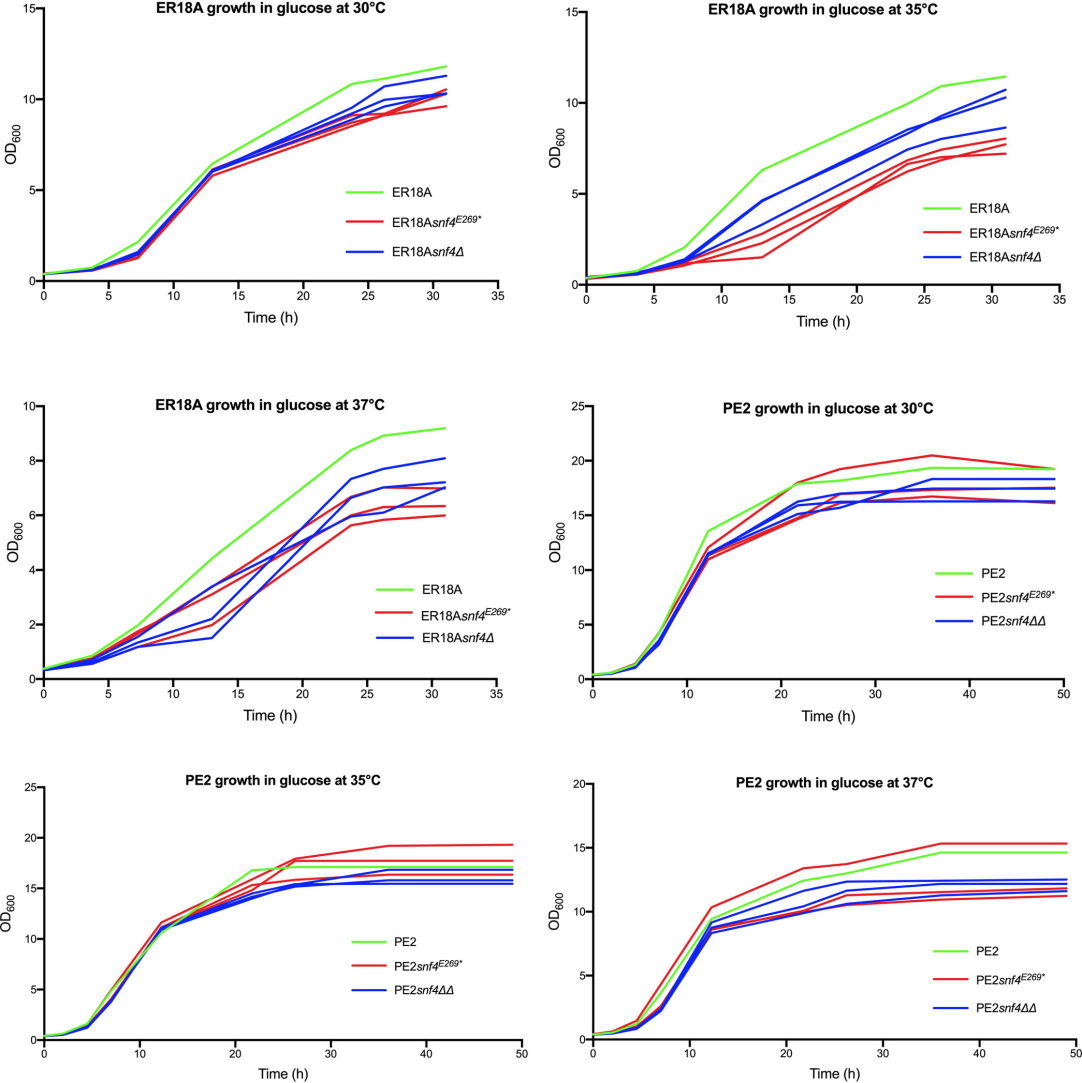
Growth assays at 30°C, 35°C, and 37°C with the ER18A and PE2 wild-type strains and derivative strains containing engineered SNP1 (*snf4*^E269^*) or *snf4Δ* in all *SNF4* alleles present, in the absence of acetic acid. The strains are indicated by different colors: ER18A and PE2 in green, ER18A and PE2 derivatives with engineered SNP1 (*snf4*^E269^*) in all *SNF4* alleles present in red (3 replicates), and ER18A *snf4Δ* and PE2 *snf4ΔΔ* strains in blue (3 replicates). Growth assays were performed in shake flasks on glucose medium.

Previous work has shown that the requirement for Snf4 is less stringent at lower temperature (23°C or 30°C) than at higher temperature (37°C), possibly due to lower levels of Snf1 protein. While in the wild-type strain the level of Snf1 protein was constant at 23°C, 30°C, and 37°C, *snf4Δ* mutants showed similar levels at 23°C and 30°C, yet strongly reduced levels at 37°C (41). Hence, we evaluated growth of the *snf4*^E269^* and *snf4Δ* strains in the two genetic backgrounds ER18A and PE2, with glucose as a carbon source. We confirm that the *snf4*^E269^* and *snf4Δ* mutations under nearly all conditions reduced the growth of the yeast and also that the requirement for Snf4 is higher at 35°C and 37°C than at 30°C. However, the latter was only true for the lab strain ER18A, while the PE2 strain showed little difference in the negative effect of the *snf4*^E269^* and *snf4Δ* mutations between these temperatures (Fig. 8).

**FIG 9:**
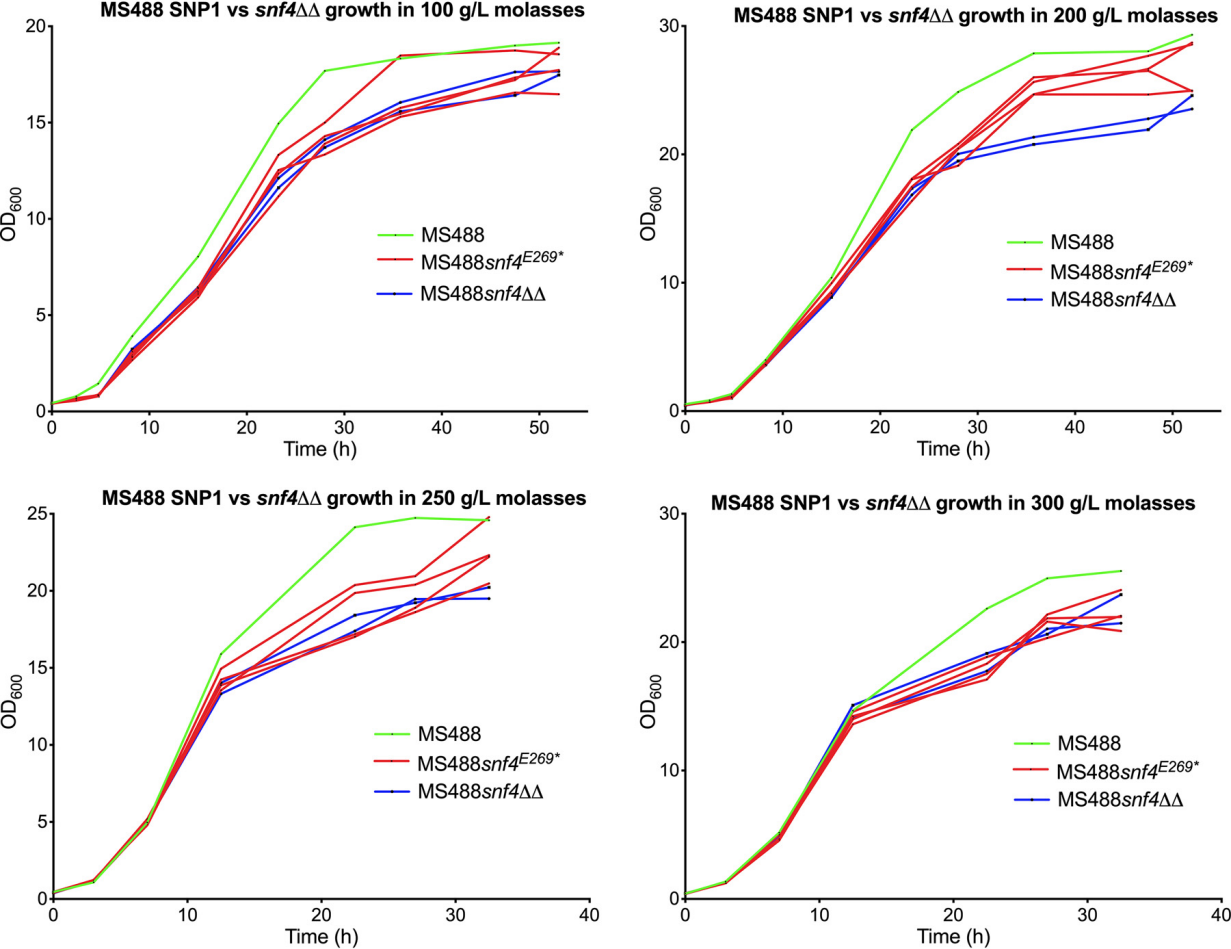
Growth of the strains MS488, MS488 with engineered SNP1 (*snf4*^E269^*) or *snf4Δ* in all *SNF4* alleles present, in four different concentrations of sugar cane molasses. The strains are indicated by different colors: MS488 in green, MS488 derivatives with engineered SNP1 (*snf4*^E269^*) in all *SNF4* alleles present in red (4 replicates), and MS488 *snf4ΔΔ* in blue (2 replicates). Growth assays were performed under aerobic conditions in shake flasks in 25 mL media with different concentrations of molasses (and absence of acetic acid) at 30°C and constant shaking at 200 rpm.

We also tested whether the *snf4*^E269^* and *snf4Δ* modifications affect the proliferation rate of the industrial strain MS488 in molasses medium by using four different concentrations of sugar cane molasses. In all cases, there was a delay in the second phase of growth and a slightly lower final optical density at 600 nm (OD_600_) (Fig. 9).

### Cumulative effect of *snf4*^E269^* or *snf4*Δ and *HAA1*^S506N^ on acetic acid tolerance.

**FIG 10:**
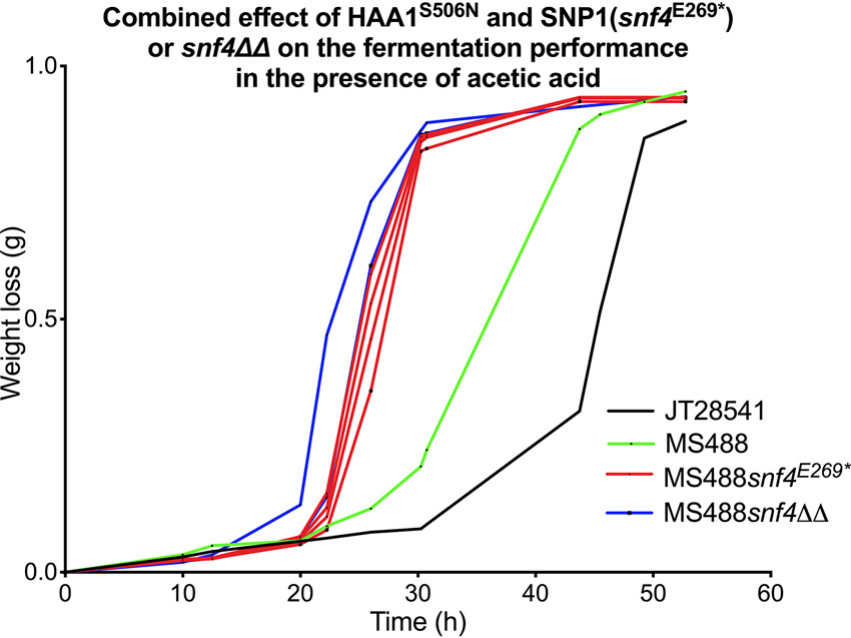
Fermentation performance of the strains JT28541 and MS488 and MS488 derivatives with engineered SNP1 (*snf4*^E269^*) or *snf4Δ* in all *SNF4* alleles, in the presence of acetic acid. The strains are indicated by different colors: JT28541 in black, MS488 (JT28541 *HAA1*^S506N^) in green, MS488 (JT28541 *HAA1*^S506N^) derivatives with engineered SNP1 (*snf4*^E269^*) in all *SNF4* alleles present in red (4 replicates), and MS488 (JT28541 *HAA1*^S506N^) *snf4ΔΔ* in blue (2 replicates). Fermentations were performed at 35°C, with constant stirring at 120 rpm, at pH 4.7 in 50 mL YPD medium with 40 g/L glucose and supplemented with 10 g/L acetic acid.

**FIG 11:**
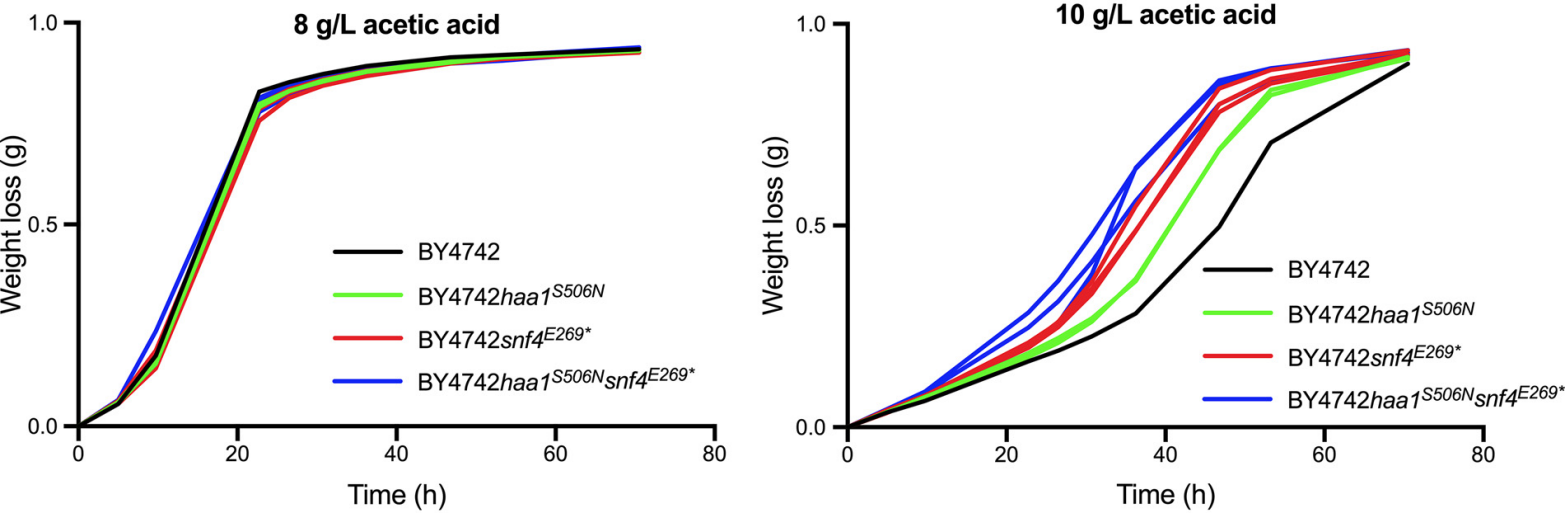
Fermentation performance of the strain BY4742 and BY4742 derivatives with engineered *HAA1*^S506N^ and/or *snf4*^E269^* in the presence of acetic acid. The strains are indicated by different colors: BY4742 in black, BY4742 *HAA1*^S506N^ in green, BY4742 *snf4*^E269^* in red, and BY4742 *HAA1*^S506N^ *snf4*^E269^* in blue. Fermentations were performed at 35°C, with constant stirring at 120 rpm, at pH 4.7 in 50 mL YPD medium with 40 g/L glucose and supplemented with 8 or 10 g/L acetic acid.

We also show that the *snf4*^E269^* and *snf4Δ* modifications were able to further enhance acetic acid tolerance in an industrial yeast strain engineered with the superior *HAA1*^S506N^ allele, which is known to cause a strong increase in acetic acid tolerance (25). For that purpose, we first engineered the superior *HAA1*^S506N^ allele in the molasses bioethanol strain JT28541, which significantly increased its acetic acid tolerance, after which introduction of the *snf4*^E269^* or *snf4Δ* modification further increased acetic acid tolerance (Fig. 10). We also engineered the *snf4*^E269^* and/or *HAA1*^S506N^ mutations in the BY4742 laboratory S. cerevisiae strain and observed a similar improvement of acetic acid tolerance for the two mutations separately and an additive effect when they were combined into a single strain (Fig. 11).

### WGT with gDNA of a strain containing NatMX and selection on medium with nourseothricin and/or acetic acid.

**FIG 12:**
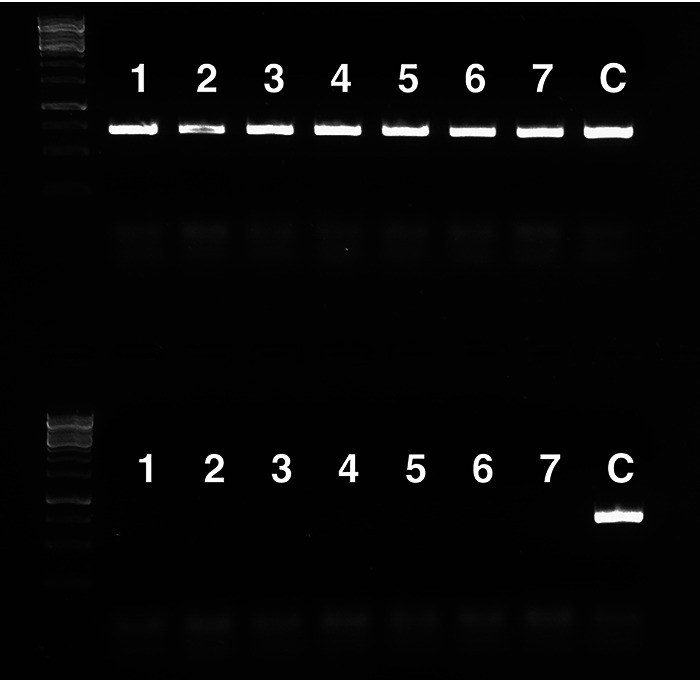
Detection of the NatMX marker by PCR amplification. (Upper row) Presence of the NatMX marker in the seven WG transformants selected on medium with nourseothricin and in the control (donor strain K11-NAT). (Lower row) Absence of the NatMX marker in the seven WG transformants after subculturing under nonselective conditions in the absence of nourseothricin.

Finally, we have tried to obtain more evidence for the possible mechanism underlying generation of the superior WG transformants with enhanced acetic acid tolerance. For that purpose, we first made use of gDNA from a yeast strain containing the NatMX marker in its genome, K11-NAT, which was constructed by deletion of the *HO* gene with the NatMX marker. After selection on medium containing nourseothricin, we obtained seven nourseothricin-tolerant WG transformants (Table 3). This indicates that with our protocol for WGT and under our conditions, fragments of the foreign genomic DNA indeed enter the host strain. The presence of the NatMX marker in all seven WG transformants was confirmed by PCR (Fig. 12). When these seven transformants were subcultured on nonselective medium (i.e., in the absence of nourseothricin), five of the seven strains completely lost their antibiotic resistance, while the two remaining strains were able to generate a small number of colonies in the presence of nourseothricin, indicating that a small part of the culture had maintained the NatMX marker (Table 3). However, with PCR amplification we were not able to detect the NatMX marker in any of the seven subcultured strains, confirming that most of the cells had lost the NatMX marker (Fig. 12). These results show that most of the WG transformants did not stably integrate the NatMX marker in the genome but likely maintained it as an extrachromosomal circular DNA (eccDNA) as long as the selective pressure remained present (i.e., in the presence of nourseothricin). The generation and maintenance under selective conditions of eccDNA have been documented repeatedly in the yeast S. cerevisiae, and there is ample evidence that such eccDNA can be transcribed (42, 43). When the WG transformants were subcultured under nonselective conditions, they all lost nourseothricin resistance, which can be explained by loss of the eccDNA containing the NatMX marker, a phenomenon that has also been documented repeatedly in yeast (42). S. cerevisiae is not able to generate spontaneous mutations that confer nourseothricin resistance, and hence no stable transformants (containing just a few SNPs, including one causative SNP, as in the case of acetic acid tolerance), could be obtained in this case. On the other hand, single SNPs are able to confer higher acetic acid tolerance in S. cerevisiae, as shown previously for *HAA1*, for instance (25), and in the present work for *SNF4*.

**TABLE 3:** Number of WG transformants obtained under selective conditions and number of stable WG transformants obtained after subculturing under nonselective conditions

Source of gDNA	Selection condition	No. of WG transformants on selective medium	No. of stable WG transformants after subculturing on nonselective medium^*a*^
No DNA (only H_2_O)	8 g/L acetic acid	53	0/20

gDNA from non-acetic acid-tolerant strain YPH499	8 g/L acetic acid	243	0/20
gDNA from non-acetic acid-tolerant recipient strain ER-18A	8 g/L acetic acid	91	1/20

Linear fragment with *HAA1*	8 g/L acetic acid	54	4/20
Linear fragment with HAA1*	8 g/L acetic acid	96	0/20

gDNA of K11-NAT	Nourseothricin	7	0/7
	8 g/L acetic acid	186	2/20
	8 g/L acetic acid + nourseothricin	0	NA

gDNA of 16D-NAT	Nourseothricin	4	NT
	8 g/L acetic acid	72	2/20
	8 g/L acetic acid + nourseothricin	0	NA

a NA, not applicable; NT, not tested.

We subsequently performed additional control experiments with WGT for enhanced acetic acid tolerance and evaluation of stability in 20 of the WG transformants obtained (Table 3). When we transformed the non-acetic acid-tolerant strain ER-18A with gDNA from another non-acetic acid-tolerant strain, YPH499, we obtained 243 transformants on selective medium, of which 0 in 20 tested were stable after subculturing. With gDNA from the host strain, ER-18A, we obtained 91 transformants, of which just 1 in 20 tested was stable. These transformants could be generated by selection and expression of a wild-type gene, like *HAA1*, which is present in the genome of any yeast strain. Indeed, when we transformed ER-18A with a linear fragment with wild-type *HAA1* or the superior mutant allele HAA1* (25), we obtained 54 and 96 transformants, respectively, of which some in the case of the *HAA1* transformants were stable. This suggests that an eccDNA can be generated from the linear fragment and maintained as long as the selective conditions are present, which gives the strain the opportunity to multiply and thus generate spontaneous mutations that confer stable acetic acid tolerance. Transformation of ER-18A with gDNA from the acetic acid-tolerant strains K11-NAT and 16D-NAT resulted in many more transformants upon selection in the presence of acetic acid (186 and 72, respectively) than upon selection in the presence of nourseothricin (7 and 4, respectively), and about 10% of the 20 acetic acid-tolerant transformants tested were stable after subculturing under nonselective conditions. Selection for combined tolerance to nourseothricin and acetic acid did not result in any WG transformants. This is likely due to the very small chance of obtaining the two required gDNA fragments in a single cell of the host strain.

## DISCUSSION

WGT has been applied most frequently and analyzed in greatest detail in bacteria. In all reports, the available evidence supports that, in this case, large DNA fragments are being incorporated by homologous recombination and that sequence similarity therefore is an important prerequisite for successful transformation. In one specific case, WGT with clinical isolates of Haemophilus influenzae, the length of the incorporated fragments was determined precisely by scoring the ±40,000 polymorphic differences between donor and recipient strains (4). They comprised 3 to 6 contiguous runs (8.1 ± 4.5 kb in length) that collectively comprised ∼1 to 3% of each transformed chromosome. In bacteria, the importance of close sequence similarity was shown for successful whole-genome transformation (5). This fits with the goal of identifying causative SNPs in antibiotic resistance genes, for which WGT has mainly been applied in bacteria (1–4).

Very few studies, on the other hand, have been reported on WGT in eukaryotic organisms. As opposed to the studies with bacteria, they were concerned with the transfer of heterologous genes conferring novel traits from the donor organism into the recipient host organism. In the first case, xylose utilization capacity and high ethanol tolerance were transferred between *P. stipitis* and S. cerevisiae by recursive WGT (6). In the second case, large gDNA fragments were transferred from wild rice to a domesticated cultivar by using selection based on an antibiotic resistance marker incorporated into the donor gDNA and phenotypic as well as randomly amplified polymorphic DNA (RAPD) characterization of the transformants (7). In both cases, large fragments of gDNA were found to be incorporated at least of the size of one or more intact genes, which conferred the required protein functionality to confer the novel phenotypic traits. The insertions therefore had to happen through random integration rather than homologous recombination (6, 7). We have made a similar observation with gDNA from a host strain carrying an antibiotic resistance marker, which resulted in antibiotic-resistant transformants carrying (at least) the complete marker gene. This shows that integration of large foreign gDNA fragments in the gDNA of the host strain does indeed happen and that such transformants can be selected under appropriate conditions. On the other hand, for complex, polygenic traits like stress tolerance characteristics, too many heterologous genes likely have to be inserted and properly expressed, and the gene products have to be functionally active and properly regulated for the whole trait to be transferred to the full extent to the host strain. This minimizes the chances that transformants displaying the complex trait could be isolated with a single WGT procedure.

In our work, we have used an acetic acid-tolerant strain and an acetic acid-sensitive strain of S. cerevisiae as gDNA donor strain and recipient strain, respectively. As a result of the previous studies in bacteria, we expected to find specific SNPs conferring high acetic acid tolerance derived from the donor strain and introduced by homologous recombination in the recipient strain. Alternatively, we might have found insertion of large fragments of the gDNA from the donor strain into the recipient strain, providing one or more causative genes conferring high acetic acid tolerance, similar to the previously reported cases in eukaryotes. Surprisingly, we found neither of the two. Instead, we found just a few novel SNPs and no other forms of genetic modification, such as duplications, indels, or chromosome copy number variations. We identified only one clear causative SNP. None of the SNPs, including the causative SNP, was present in the gDNA of the donor strain. On the other hand, the gDNA of the superior donor strain was essential since transformation with the host strain gDNA, with gDNA from an S. cerevisiae strain with similar acetic acid tolerance, with random DNA, or with water never resulted in any stable transformants with higher acetic acid tolerance. The present results are consistent with those of two other projects that we recently completed in our group and in which WGT was used to generate strains with improved thermotolerance or improved HMF tolerance (8, 9). Also in these cases, a single causative SNP was identified among just a few SNPs present in the WG transformants, without any foreign DNA being detectable. The causative SNP was also absent from the donor genome, and transformation with DNA from nontolerant strains, random DNA, or simply water never resulted in stable, superior transformants. The striking similarity between the results of the three WGT projects points to a reliable methodology with a predictable and reproducible outcome, which should find general use and application for the improvement of selectable traits in industrial yeast strains. On the other hand, the unexpected outcome raises intriguing questions as to the mechanism involved in generating these superior WG transformants.

A possible hypothesis is that a linear fragment of donor gDNA containing a causative gene for high acetic acid tolerance may have entered the host strain and propagated for some time (e.g., after circularization into eccDNA), supporting the survival of the host strain as long as the selective stress condition of high acetic acid remains present. Heterologous DNA in the form of eccDNA plasmids is unstable and gets randomly lost during proliferation. Once the eccDNA is lost, the cells cease proliferation. However, the cells of the WG transformant that maintain the eccDNA can continue proliferation at least for some time until they lose the plasmid. The continued proliferation of the WG transformant’s cells that maintain the eccDNA allows the cells to generate random spontaneous mutations, including mutations that by coincidence confer high acetic acid tolerance. Hence, the presence of the protecting element on the eccDNA facilitates the selection of spontaneous mutations in the host strain during its further proliferation that fortuitously confer high acetic acid tolerance. Once such a mutation is present, the eccDNA can be lost without impeding the further proliferation of the cells of this mutant. All descendants of this mutant will now be able to continue proliferation and can also never regain the eccDNA. On the other hand, among the transformants that do not generate in time a protecting mutation, there will always be descendants that lose the eccDNA and thus cannot continue proliferation. Hence, the proliferation of eccDNA transformants without the protecting mutation will always be much less than 100%. As a result, after prolonged cultivation the whole culture will consist of spontaneous mutants with a protecting mutation and lacking the eccDNA. Different possibilities exist for the type of heterologous gDNA element on the eccDNA. It may be limited to a very small fragment, like a promoter region, titrating out a transcriptional repressor in the host strain. We also have no evidence that the protection by the heterologous element on the eccDNA and the protection by the spontaneous mutation in the host strain have an additive effect against acetic acid stress. This would probably require much more sensitive tests than the growth and fermentation tests we have performed.

Entry of gDNA fragments in the host cells may have been a frequent phenomenon, but only transformants with a fragment conferring high acetic acid tolerance would be able to survive the selective condition, and only those generating in time a stable, spontaneous mutation in the genome conferring high acetic acid tolerance would have been able to permanently survive the selective condition. This hypothesis can explain why none of the new SNPs in the host strain transformant, including the causative SNP, was present in the donor gDNA. The hypothesis could be experimentally verified by large-scale transformation of yeast strains with gDNA from many other yeast species and selection under different stress conditions. If a correlation would appear between the tolerance of the gDNA donor strains against a specific type of stress and the success of isolating transformants in the host strain under the same stress condition, it would provide support for this model. Although we have not done an exhaustive analysis yet in this respect, the recent isolation of thermotolerant and HMF-tolerant WGT transformants in S. cerevisiae in both cases resulted in the same situation as described in this article and supports this model (8, 9). All SNPs in the gDNA of successful transformants, including the causative SNPs, were never present in the donor gDNA, and transformation with gDNA from nontolerant species or strains never resulted in stable transformants with an improved phenotype (8, 9). An alternative hypothesis might be that the gDNA from the donor strain acted in some way as a general mutagen in the host strain, but this appears unlikely in view of the observation that random DNA or gDNA from a nontolerant strain, including the host strain's own DNA, was ineffective in generating stable transformants with enhanced acetic acid tolerance. Transformation with water also never resulted in stable strains with an improved phenotype. All these data contradict that the causative mutation was already accidentally present or generated spontaneously before the WGT procedure was applied.

In order to prove the presence of a heterologous DNA element on eccDNA in the host strain, special precautions will have to be used to maintain the eccDNA in the host strain. After WGT, only a single cell contains the eccDNA, and to identify the transformant, it has to grow up into a visible colony on agar nutrient medium containing a high acetic acid concentration. By the time there is a visible colony, at least in the current WG transformants, the putative eccDNA is lost because the spontaneously generated *snf4*^E269^* mutation has taken over the protection against the high acetic acid stress. Hence, the eccDNA cannot be recovered anymore from the visible colony. On the other hand, the additional experiments that we performed with the NatMX marker provide indirect evidence for the presence of an eccDNA element. When we used genomic DNA from a donor strain with the NatMX marker and selected WG transformants on medium with nourseothricin, we were able to isolate transformants growing into visible colonies in which we could demonstrate the presence of the NatMX marker by PCR. Moreover, the marker got easily lost after subculturing on nonselective medium without nourseothricin, indicating that it did not get inserted into the genome but must have been present in an unstable form. Since linear DNA fragments cannot be maintained as such in yeast cells, these results strongly point to the presence of the NatMX marker in the form of an unstable eccDNA element in the host strain.

Whatever the underlying molecular mechanism for generation of the WGT transformants, it raises unexpected questions with respect to the genetically modified organism (GMO) status of the resulting strains. WGT is a natural process that appears to have occurred frequently in evolution; it may also be considered a classical mutagenesis technology, making it exempt from the GMO regulations as applied in Europe. The generation of spontaneous mutations clearly leads to organisms not subject to GMO regulations. On the other hand, the introduction of heterologous DNA in a host strain is a major argument for application of GMO regulation. Hence, the current WGT transformants seem to have mixed characteristics of cisgenic and transgenic organisms: they appear to be “transiently transgenic and permanently cisgenic.” This may raise a dilemma for application of current GMO regulations in Europe and many other countries (44, 45).

The number of SNPs, especially the number of nonsynonymous SNPs, was surprisingly low in the transformants. This may in part have to do with the haploid nature of the host strain. Deleterious mutations may have been filtered out, while in a diploid strain, deleterious mutations may be kept in heterozygous form. On the other hand, if our hypothesis is correct that the new SNPs in the transformants are generated by spontaneous mutagenesis under transient protection of a genetic element on an unstable fragment of the donor gDNA, it would explain why the number of new mutations in the WGT transformants is so low. A drawback of classical mutagenesis or evolutionary adaptation for industrial strain improvement is the large number of background mutations that are generated in the strains (46). These often cause unexpected side effects on industrially important properties, especially in stages of the industrial application, which are generally not evaluated at lab scale, such as the industrial production conditions of the bulk yeast or the drying tolerance of the yeast (47–49). Elimination of background mutations is much more cumbersome in industrial yeast strains compared to laboratory strains. Industrial yeast strains, for instance, are generally heterothallic, diploid, polyploid, and/or aneuploid, and isogenic series of strains displaying the same range of commercially important properties are not available (47–49). WGT could be an efficient strategy to overcome this problem by offering a means for rapidly generating and identifying superior alleles capable of improving specific selectable traits of interest or for directly generating superior variants of industrial yeast strains with a minimal number of background mutations.

Given the unexpected outcome of a single causative mutation appearing in the host genome as a result of the WGT approach using heterologous DNA, a plausible question can be raised as to the possible advantage of this approach compared to classical mutagenesis with, for instance, ethyl methanesulfonate (EMS) or UV. In the latter case, however, intensive treatment with the mutagen is generally applied to maximize the chances of successful isolation of a rare mutant with the desired phenotype. This generally results in a reduction of viability in the mutagenized culture, with a minority of cells surviving the treatment. The surviving strains generally contain hundreds of mutations, of which one or more are causative for the selected phenotype of interest, but several other mutations may negatively affect other properties of the yeast. For basic research on lab strains, this generally does not present a major problem, but for industrial strains, a single negative effect on an industrially important trait can easily render the strain useless for industrial application. On the other hand, application of a small dose of EMS or UV to limit the number of mutations introduced in the genome to just 5 to 10, as in the case of the WGT procedure, is generally ineffective for isolating mutants with a superior phenotype, because of the minute or even negligible chances of generating a beneficial mutation in the whole population for the phenotype under selection. Moreover, when selecting for mutants with further increased tolerance to a specific stress condition in industrial strains that already display a high level of intrinsic tolerance to the stress condition, the chances of isolating superior strains are even more limited because of the scarcity of the remaining beneficial mutations and the high chances of negative side effects when many additional random mutations are introduced.

The whole-genome transformation methodology is a gentler mutagenesis procedure since it apparently relies on spontaneous mutations generated in the host genome. The number of such mutations is much lower than the number generated by the harsh classical mutagenesis procedures, but the proliferation of the transformants under protection of the heterologous DNA is apparently long enough to generate extensive progeny displaying a broad portfolio of spontaneous mutations, each carrying just a few mutations in a single offspring strain. Spontaneous mutations that compromise the phenotype will be eliminated anyway in the selection procedure. Moreover, in classical mutagenesis, there is just a single shot of mutant generation, in which the desired beneficial mutation has to be established immediately. On the other hand, in the whole-genome transformation and selection procedure, at least when our interpretation is correct, new mutants are generated continuously during the proliferation of the transformed host cells, as well as their daughter cells, during multiple generations in an exponential fashion, which increases the chances of generating the desired beneficial mutation. Hence, to generate superior mutations in industrial strains that cause a further improved selectable phenotype, whole-genome transformation might be more effective than classical mutagenesis.

Our results provide strong evidence that the nonsense *SNF4* mutant allele, encoding the truncated Snf4 protein, had the same effect on increasing acetic acid tolerance as deletion of the *SNF4* gene. This was observed in all genetic backgrounds tested. To the best of our knowledge, this is the first time that the *SNF4* gene has been implicated in tolerance against acetic acid. Since Snf4 is known to be involved in the glucose repression pathway, inactivation of the gene will likely affect other properties of the yeast. These side effects are not necessarily negative in industrial applications. We noticed that inactivation of *SNF4* caused a reduction in the growth rate, which might be negative for industrial propagation of the yeast, but could be positive in industrial fermentations because it could lower biomass formation. Introduction of additional mutations might also alleviate the side effects while maintaining the beneficial effect on acetic acid tolerance.

It remains unclear how the inactivation of *SNF4* enhances acetic acid tolerance. Snf4 is an activating subunit of Snf1, and it binds to the carboxy-terminal regulatory domain of Snf1. Interaction between Snf1 and Snf4 is inhibited in the presence of high levels of glucose (50). In laboratory strain backgrounds, *snf4Δ* mutants are unable to grow on maltose or on nonfermentable carbon sources (40, 51). Although we also noticed slight growth delays on some carbon sources in the *snf4* inactivation mutants, these effects varied with the genetic background of the strain and were far less dramatic than would have been expected from the reports with laboratory strains. In the industrial strain backgrounds, the function of Snf4 appears far less essential for properties controlled by the main glucose repression pathway, explaining why we unexpectedly isolated an *snf4* inactivation mutation in a screen for strains showing better growth under stress. Since the stability of Snf1 was reported to be lower at higher temperatures (41), we also checked growth of the *snf4* mutants in two different strain backgrounds at 30°C, 35°C, and 37°C. We could only detect a stronger growth reduction at higher temperatures in the lab strain ER18A and not in the PE2 strain, again supporting that the function of Snf4 in the industrial strain backgrounds is much less stringent. It has been shown that the regular temperature range of 3 to 42°C for growth of S. cerevisiae is narrowed to 19 to 26°C in the presence of 1% (vol/vol) acetic acid (52). Although we found that 35°C is the optimal fermentation temperature of ER18A in the absence of acetic acid, in its presence, the temperature of 35°C that we used in all fermentation experiments could have acted as an additional stress factor. This might be true also for other factors, such as higher ethanol sensitivity in the presence of acetic acid. Hence, the higher sensitivity to other stress factors can be considered as part of the toxicity caused by acetic acid. The *snf4*^E269^* and *snf4Δ* mutations’ protecting effect could thus be directed in part against increased temperature stress and/or one or more additional intensified stress factors. Since Snf4 is involved in the glucose repression pathway, it could, also in the industrial strain backgrounds, lower the level of expression of acetic acid transporters like Jen1 and Ady2 or that of the Fps1 channel protein, which mediates acetic acid uptake from the medium, or enhance the expression of acetic acid efflux pumps (53). Alternatively, it could help stabilizing the intracellular pH by increasing the expression level of the plasma membrane H^+^-ATPase gene, *PMA1* (12). The results shown in Fig. 6 appear to contradict that *snf4Δ* stimulates the total consumption of acetic acid, although it appears to stimulate the initiation of acetic acid utilization.

Genetic analysis of natural strains and different types of mutants, including gene deletion and overexpression strains, has been revealing an ever-increasing number of genes and alleles than can enhance acetic acid tolerance (19–21, 25–29, 33). We have now shown that combination of *snf4*^E269^* or *snf4Δ* with *HAA1*^S506N^ in a single strain provides further improvement of acetic acid tolerance. This raises the interesting question of to what extent acetic acid tolerance can be enhanced by accumulation of many different mutant alleles present in natural strains or obtained by mutagenesis. The much higher acetic acid tolerance of Z. bailii shows that yeast cells are at least in some way able to tolerate much higher acetic acid levels than the regular S. cerevisiae strains (36). Whether this is due to modified expression or activity of components also present in S. cerevisiae or is due to novel gene products in Z. bailii remains unclear (36, 37). Very little is known about the function of the second gene, *YJR120W*, in which we identified an SNP that affected the phenotype. Deletion of *YJR120W* causes strong phenotypic effects, including an inability to grow under anaerobic conditions, decreased expression of *ATP2*, impaired respiration, defective sterol uptake, and altered levels and/or localization of ABC transporters Aus1 and Pdr11 (54–56). It has been suggested that *YJR120W* is not a functional gene itself and that the effects of its deletion are only due to a neighboring gene effect on the expression of *ATP2* (57).

In conclusion, we have shown that WGT of an S. cerevisiae strain with gDNA from an S. cerevisiae strain with much higher acetic acid tolerance allows the isolation of transformants with clearly enhanced acetic acid tolerance. Unexpectedly, the transformant showed a very low number of new SNPs, none of which was present in the donor strain gDNA. The new SNPs in the transformant might have been generated by spontaneous mutagenesis under transient protection of a genetic element in an unstable fragment of the donor gDNA (e.g., maintained as eccDNA in the host cell). The causative SNP was a nonsense and inactivating mutation in *SNF4* that caused only slight side effects on other phenotypes in industrial strain backgrounds compared to what would have been expected from the literature on *SNF4* in laboratory strain backgrounds. This makes this *snf4*^E269^* allele an interesting tool for improvement of acetic acid tolerance in industrial strains by cisgenic genetic modification.

## MATERIALS AND METHODS

### Yeast strains and media.

The S. cerevisiae strains used and constructed in this work are shown in Table 4. In most experiments, YP medium (10 g/L yeast extract, 20 g/L bacteriological peptone) was used, supplemented with different concentrations of glucose. In addition, media with different concentrations of molasses were used, as indicated. Yeast propagation was done in YP with 20 g/L glucose, while fermentation and growth assays were done in YP supplemented with 40 g/L glucose. Propagation was performed in a shaking incubator at 30°C with constant shaking at 200 rpm. For selection of transformants, solid YP medium was used, containing 20 g/L glucose and 15 g/L Bacto agar and supplemented with different concentrations of acetic acid, without or with pH correction to 4.7 using 4 M KOH. The pH was corrected to 4.7, which is just below the pK_a_ of acetic acid (4.76), to ensure stringent conditions.

**TABLE 4:** S. cerevisiae strains used and constructed in this work

Strain	Origin or genotype	Source or reference
Ethanol Red (ER)	First-generation (corn, wheat) bioethanol production strain	Fermentis (a division of S. I. Lesaffre, France)
ER18A	Segregant of ER	25
JT28541	Molasses bioethanol production strain	Lab strain collection
K11	Sake production strain	Lab strain collection
K11-NAT	K11 strain containing NatMX	This study
PE2	Brazilian (sugar cane) bioethanol production strain	Lab strain collection
MS164	WGT of ER18A	This study
MS171	ER18A + SNP1^MS164^	This study
MS172	MS164 + SNP1^ER18A^	This study
MS173	ER18A + SNP2^MS164^	This study
MS174	MS164 + SNP2^ER18A^	This study
MS175	ER18A + SNP3^MS164^	This study
MS176	MS164 + SNP3^ER18A^	This study
MS177	ER18A + SNP4^MS164^	This study
MS178	MS164 + SNP4^ER18A^	This study
MS179	ER18A + SNP5^MS164^	This study
MS180	MS164 + SNP5^ER18A^	This study
MS181	ER18A + SNP6^MS164^	This study
MS182	MS164 + SNP6^ER18A^	This study
MS183	ER18A + SNP7^MS164^	This study
MS184	MS164 + SNP7^ER18A^	This study
MS488	JT28541 *HAA1^S506N^*	25; this study
MS493	PE2 + SNP1^MS164^	This study
MS500	PE2 *snf4*△△	This study
MS608	ER18A *snf4*△	This study
MS649	MS488 + SNP1^MS164^	This study
MS653	MS488 *snf4*△△	This study

As the inferior host strain for WGT, we used ER18A, a haploid segregant derived from Ethanol Red, an industrial yeast strain used in commercial first-generation bioethanol production. The strain has a high robustness and fermentation capacity, yet relatively low tolerance to acetic acid. As a gDNA donor, we have used K11, a sake strain with high acetic acid tolerance. To evaluate the beneficial effect of the newly discovered genetic modifications in different strain backgrounds, we have used PE2, an industrial strain used in Brazil for bioethanol production with sugar cane, and MS488, an industrial strain developed for bioethanol production with molasses.

### Screening of the yeast strain collection.

To identify the strain most suitable as a donor of the gDNA, we screened all S. cerevisiae strains with a known genome sequence for high acetic acid tolerance by spot assays in which a culture with OD_600_ of 1.0 was used, 1:10 serial dilutions were spotted onto YPD nutrient plates in the presence of different acetic acid concentrations from 6 g/L to 10 g/L at pH 4.7, and growth was evaluated after incubation of the plates at 30°C. From this evaluation, we selected the K11 sake strain as the strain with the highest acetic acid tolerance. This strain lacked all the superior alleles for high acetic acid tolerance we identified in our previous study (25) and thus was expected to contain novel causative alleles for high acetic acid tolerance. The screening was done on solid YPD medium supplemented with different concentrations of acetic acid ranging from 4 g/L to 6.5 g/L, without pH correction. The K11 strain was able to grow up to 6 g/L acetic acid (without pH correction).

### Genomic DNA extraction and whole-genome transformation.

High-quality gDNA was extracted using the MasterPure yeast DNA purification kit from Epicentre according to the manufacturer’s instructions. The gDNA fragments obtained were not cut further into smaller pieces for whole-genome transformation.

An overnight culture of the host strain, ER18A, was transformed with 2 μg of donor gDNA using the standard electroporation protocol (58), which uses dithiothreitol (DTT), LiAc, and sorbitol. The electroporation was performed on the yeast culture mixture in electroporation cuvettes with the pulse settings 1,500 V, 25 μF, and 200 Ω. After transformation, a standard 4-h recovery was carried out in nonselective YPD medium. The transformed culture was then plated on solid YPD medium containing a range of acetic acid concentrations, from 5 g/L to 10 g/L, with pH corrected to 4.7 using 4 M KOH, and grown for 2 days at 30°C. All colonies obtained on the medium with the highest concentration of acetic acid (10 g/L) were tested in spot assays on YPD solid medium with 6 g/L to 10 g/L acetic acid at pH 4.7 at 30°C. For this purpose, the cells were first grown until stationary phase in liquid YPD medium at 30°C, and the starting OD_600_ was adjusted to 1, followed by 1:10 serial dilutions for the spot assays. The transformant with the highest acetic acid tolerance, selected in this way, was called MS164.

### Whole-genome sequence analysis and identification of SNPs.

The gDNA of MS164 and ER18A was extracted with the MasterPure yeast DNA purification kit (Epicentre) and sent to the Beijing Genomics Institute (BGI; Hong Kong) for whole-genome sequence analysis. A library of 125 paired-end reads with an average insert length of 500 bp was generated with the Illumina HiSeq2500 platform. Reads of strain K11 were obtained from NCBI (accession no. SRR1568238). All reads were mapped against the S288c reference genome (version R64-2-1; SGD) with bowtie2, using parameters -I 0 -X 600 -a -t. Variant detection was performed with NGSEP (59), using parameters -maxBaseQS 30 -minQuality 40 -maxAlnsPerStartPos 2. Repetitive regions were masked using Tandem Repeats Finder (60). The final VCF file was filtered with parameter -q 40 and annotated. Custom in-house scripts were used to extract variants introduced in MS164 that did not occur in ER18A. These variants were then compared to the corresponding sequence in K11 to determine whether these variants could have been derived from K11 or were novel mutations introduced during the transformation of ER18A with the gDNA from K11.

Unmapped reads were collected and *de novo* assembled using CLC Genomics Workbench (Qiagen Bioinformatics) with default parameters. These contigs were used as a reference, and a similar mapping strategy was followed, after which the variants of MS164 and ER18A were compared again. All contigs of MS164 were BLAST searched against the K11 genome to identify possible large-scale insertions. Additionally, copy number variations were analyzed with in-house scripts to identify possible duplications/deletions or large-scale genome rearrangements.

### CRISPR/Cas9 technology.

We have used clustered regularly interspaced short palindromic repeat (CRISPR)/Cas9 technology (61–64) to transfer SNP1 into strains with different genetic backgrounds by using the following steps.

(i) Cas9 plasmid transformation was done with the P51 (pTEF-Cas9-KANMX) single-copy plasmid derived from the p414-TEF1p-Cas9-CYC1t plasmid (62). Transformants were selected on YPD plates containing geneticin. Transformation was done with 1 μg of Cas9 plasmid using the LiAc–single-stranded carrier DNA–polyethylene glycol (PEG) method (65, 66).
(ii) For gRNA plasmid transformation, a specific gRNA targeting SNP1 was designed. gRNA was flanked by GCAGTGAAAGATAAATGATC (promoter) and GTTTTAGAGCTAGAAATAG (terminator) and without a protospacer adjacent motif (PAM) site. Both forward and reverse oligomers were ordered, duplexed, and assembled by the Gibson Assembly kit in the XhoI-EcoRV-digested P58 vector. P58 contains the HPH marker in the p426-SNR52p-gRNA.CAN1.Y-SUP4t backbone (62). Forward and reverse oligomers at 500 μM dissolved in STE buffer (10 mM Tris [pH 8.0], 50 mM NaCl, 1 mM EDTA) were duplexed by incubating equimolar concentrations of the primers for 3 min at 94°C and slowly cooling down by turning off the heat block.
(iii) For the SNP1 replacement, we used 59-bp oligonucleotide donor DNA containing the desired SNP1: the forward (AGAAGCCCTTATGAGGAGAAGTGATGATTTTTAAGATGTTTATACATGCACTAAGAATG) and reverse (CATTCTTAGTGCATGTATAAACATCTTAAAAATCATCACTTCTCCTCATAAGGGCTTCT) oligonucleotides were duplexed using the same protocol described for gRNAs.

A successful Cas9 transformant was afterwards transformed with 1 μg of gRNA plasmid and 2 μg of donor DNA using the LiAc–single-stranded carrier DNA–PEG method. The transformation mixture was plated on solid YP medium with 20 g/L glucose supplemented with 200 μg/mL Geneticin and 300 μg/mL hygromycin B.

The gRNA plasmids for application of the CRISPR/Cas9 technology were amplified in Escherichia coli cells grown overnight at 37°C on solid Luria broth (LB) medium with 15 g/L Bacto agar and the respective antibiotic.

### Targeted gene deletion.

Depending on the ploidy of the strain, the *SNF4* gene was deleted in one or two copies. Deletion cassettes were amplified using the plasmids with NatMX and KanMX antibiotic markers with 60-bp flanking regions for homologous recombination. The amplification was done by PCR using Q5 enzyme. Two different PCRs were performed to verify whether the *SNF4* gene deletion was correct: the first PCR comprised primers binding inside the antibiotic marker and outside the gene, while the second PCR comprised primers inside and outside the gene. If we got a positive result for the first PCR and a negative result for the second, we concluded that deletion of the gene was correct.

### Allele-specific PCR and Sanger sequencing.

To verify correct SNP1 replacement, we first performed allele-specific PCR. The forward primer differed in a desired SNP at the 3′ end, one being specific for ER18A (ATGAGGAGAAGTGATGATTATG) and the other for MS164 SNP (ATGAGGAGAAGTGATGATTATT). Both primers contained an extra mismatch at the third nucleotide position from the 3′ end to increase specificity. The reverse primer (GCCTGTACCTTTTTGATG) was common for both PCRs and was designed to be at a distance of about 500 bp. Depending on whether SNP1 was present, only one of the two PCRs gives a positive result. When a transformant with the correct mutation was identified, it was grown with several transfers in liquid YPD in order to lose the Cas9 and gRNA plasmids. Single cells were picked using a micromanipulator, and the strains were sent for final verification by Sanger sequencing.

### Small-scale fermentations.

Whole-genome transformants, SNP1 replacement strains, and gene deletion strains were evaluated for acetic acid tolerance under semianaerobic fermentation conditions. The medium used was YP containing 40 g/L glucose and various concentrations of acetic acid, with initial pH adjusted to 4.7 using 4 M KOH. Yeast cells used were pregrown in YP with 20 g/L glucose for 48 h at 30°C until stationary phase. After measuring the OD_600_ of each culture, the correct volume needed was calculated, and the cells were harvested by centrifugation at 3,000 rpm for 5 min at 4°C. The starting OD_600_ of the fermentations was 2, corresponding to approximately 0.5 g cell dry weight (CDW)/L cell density. Small-scale semianaerobic fermentations were performed in a 50-mL volume at 35°C with continuous stirring at 120 rpm. Fermentation performance was assessed by measuring the weight loss of the fermentation tubes, which corresponds to CO_2_ release and correlates directly with ethanol production because of the glucose repression of respiration and the semianaerobic conditions during the fermentations. Due to the differences in strain sensitivity, the ER18A and MS488 strains were tested at 10 g/L acetic acid, while for PE2, we used 11 g/L acetic acid, both at pH 4.7. Under our conditions (50 mL, 40 g/L glucose), the maximum weight loss was 1 g.

### Growth assays.

Growth assays of the strains were done in shaking flasks under aerobic conditions. The medium used was YP containing 40 g/L glucose and 10 g/L acetic acid, with pH adjusted to 4.7 using 4 M KOH. Growth tests were done with a starting OD_600_ of 2 in flasks containing 50 mL of the culture in a shaking incubator at 30°C and with constant stirring at 200 rpm. To evaluate the utilization of different carbon sources, we performed screening using a Multiskan FC microplate photometer (Thermo Fisher), with a starting OD_600_ of 0.1 in 200 μL YP medium containing 40 g/L glucose, maltose, or sucrose. Growth was measured at 30°C and 37°C with pulsed stirring of 1 min every 15 min. Additionally, we evaluated the extent of aerobic growth in flasks containing 25 mL of the culture in 100 g/L and 200 g/L molasses with a starting OD_600_ of 0.5 in a shaking incubator at 30°C and with constant stirring at 200 rpm.

### High-performance liquid chromatography.

During the growth assays in shaking flasks, as well as during the fermentations in YP with 40 g/L glucose, samples were taken every few hours to assess glucose utilization, ethanol production, and the level of acetic acid. Samples were analyzed using a Shimadzu Nexera X2 HPLC system. An H_2_SO_4_ concentration of 5 mM was used as the mobile phase, with a flow rate of 0.7 mL/min over an Agilent MetaCarb 87H column (300 by 7.8 mm) at 70°C. The compounds were detected using refractive index detection and analyzed using LabSolutions software (version 5.86; Shimadzu Corporation).

### Reproducibility of the results.

The data show representative results of two complete repetitions using the number of biological replicates indicated in the figure legends, and giving consistent results. All biological repeats are shown individually in the figures.

### Availability of data and materials.

All data have been stored on dedicated computers at KU Leuven. All data and yeast strains are freely available upon request.
